# Treatment of Atrophic Facial Scars Using Polydioxanone Threads: A Case Series

**DOI:** 10.7759/cureus.63403

**Published:** 2024-06-28

**Authors:** Mohammad Khaled Hamolaila, Mazen Zenati, Mohammad Y Hajeer

**Affiliations:** 1 Department of Oral and Maxillofacial Surgery, Faculty of Dentistry, University of Damascus, Damascus, SYR; 2 Department of Orthodontics, Faculty of Dentistry, University of Damascus, Damascus, SYR

**Keywords:** vertically oriented scars, facial appearance, forehead scar, scar appearance, non-surgical techniques, polydioxanone threads, atrophic facial scars

## Abstract

The appearance of scars affects patients' aesthetic and psychological aspects, as atrophic scars can result from previous surgeries or inflammatory/infectious conditions. Recently, non-surgical techniques have been introduced to improve scar appearance and enhance patient satisfaction. To our knowledge, there has been limited published medical research evaluating the effectiveness of polydioxanone threads in managing facial scars. This report aims to present three cases where scars were managed using these materials in the facial area with a follow-up of six months post-intervention. Based on the three presented cases, it is shown that there was an improvement in the color and texture of the scar, in addition to its reduced size with no sensation of pain or itching after the procedure. These findings suggest that the materials used are promising for effectively treating facial scars.

## Introduction

Wound healing is a complex and tightly regulated biological process consisting of three interrelated stages: the inflammatory stage, the cellular proliferation stage and the tissue remodeling stage [1]. The remodeling stage is marked by reduced cellular activity and decreased blood vascularization due to programmed cell death, and this phase involves increased synthesis and deposition of the extracellular matrix at the injury site, leading to the formation of a mature skin scar in adults [1]. The primary goal in managing visible pathological scars is to improve aesthetics, enhance patient confidence, elevate quality of life, and reduce symptoms like pain and restricted mobility [2]. Facial scars are managed through preventive and corrective methods to prevent formation and remove existing scars [2]. Corrective procedures for managing skin scars include non-invasive techniques characterized by their ease of application, such as hyaluronic acid injections or corticosteroid injections with minimal patient impact [3]. Additionally, invasive surgical techniques aim to excise pathological scars and remove them along with the surrounding affected tissues [3].

Polydioxanone (PDO) is marketed commercially as monofilament threads under the name PDS®, and approved for use by the US Food and Drug Administration (under number K190264). PDO threads are more elastic than polypropylene threads and exhibit greater tensile strength. They elicit less intense tissue reactions and are safely absorbed via hydrolysis [4]. PDO threads are used in the cosmetic field for skin lifting and to improve skin quality [5]. However, despite their healing qualities and role as a dermal filler, they did not get fame in scar management. In light of the previous, using PDO threads is a low-cost and an acceptable method [6]. However, relatively few cases document its application for scar improvement compared to the extensive literature on its use for reducing signs of aging [7]. This article presents a report on several facial atrophic scars managed using PDO threads with a follow-up of six months post-surgery.

## Case presentation

This case series report presents three cases of patients who visited the Department of Oral and Maxillofacial Surgery Hospital at the Faculty of Dentistry at Damascus University. These three cases shared the presence of a single visible scar in the forehead area.

Case 1

**Figure 1:**
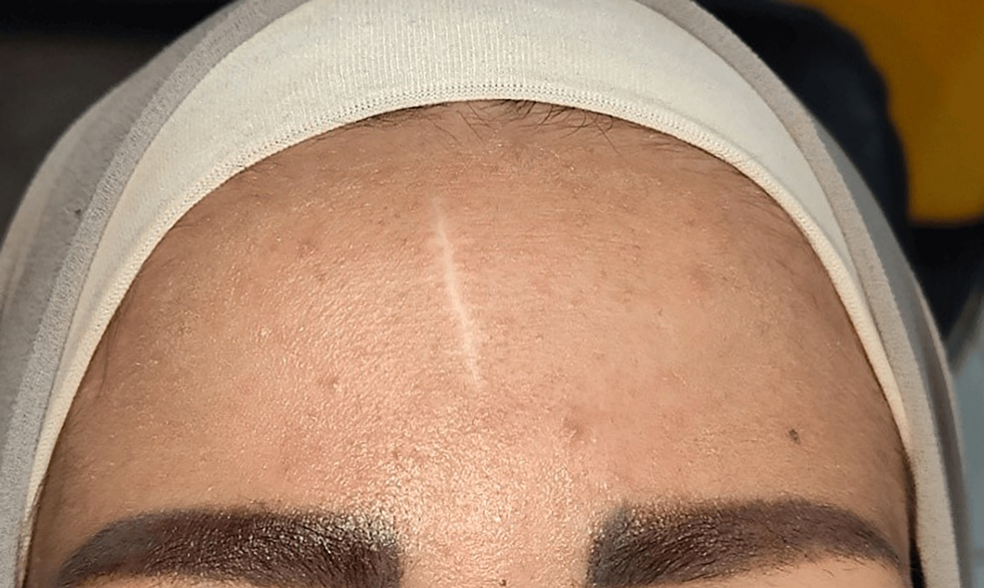
A close-up view of the forehead scar before starting the procedure

A 22-year-old female patient presented with an atrophic scar on the forehead of four years due to a car accident, with no prior treatment for the scar (Figure 1).

**Figure 2:**
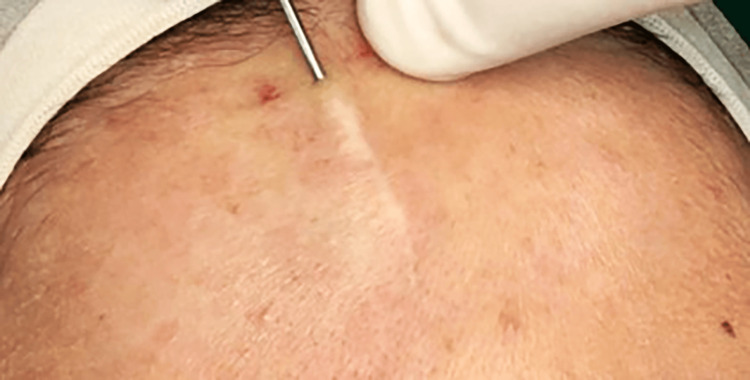
The entry point of the thread

After explaining the treatment plan and the material used, the patient’s written consent was obtained for the proposed treatment using polydioxanone threads; this choice was made due to the type of scar and its linear shape. First, the skin was disinfected at the surgical site using a 4% povidone solution; skin anesthesia at the surgical site was achieved using the 20% benzocaine topical anesthetic, and local anesthesia was administered by injecting 2% lidocaine with 1:80,000 epinephrine around the scar edges. The surgical site was isolated using sterile surgical drapes, and the surgical operating table was prepared. Using an 18-gauge needle, an entry point was made at the beginning of the scar, and the needle was inserted along the scar length to its end to create a passage for inserting the needle carrying the polydioxanone threads, as shown in Figure 2.

**Figure 3:**
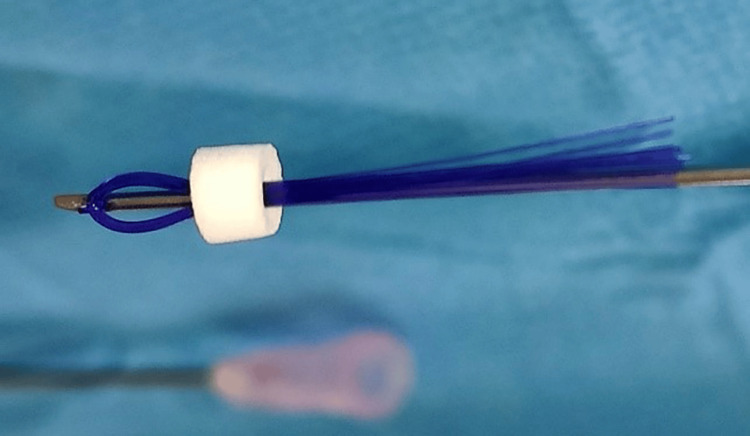
Polydioxanone threads used in the presented case

A needle carrying the threads (with a non-cutting head) loaded with 14 threads was then inserted. Smooth type PDO threads (Hydra Multi; BeauMed, Republic of Korea) measuring 50 mm for the thread and 38 mm for the needle were used, with a non-cutting head needle with 14 strands of polydioxanone size 0-7, with a main thread, as shown in Figure 3.

**Figure 4:**
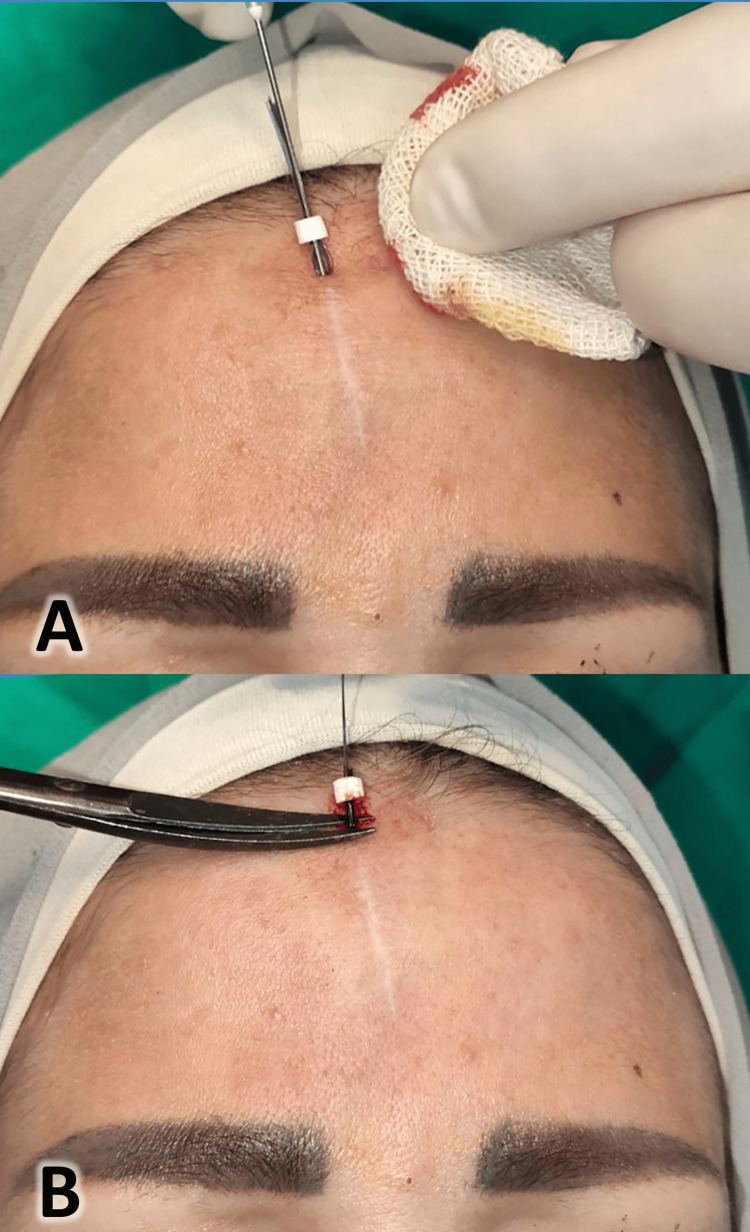
Insertion of the polydioxanone threads into the scar (A) and trimming of the excess thread (B)

The subcutaneous layer was the targeted layer for thread placement; the cannula was inserted with gentle pressure by the left hand on the scar; the cannula (needle carrying the threads) was removed gently, leaving the threads in place under the skin, and the excess part of the threads was trimmed, as shown in Figure 4.

**Figure 5:**
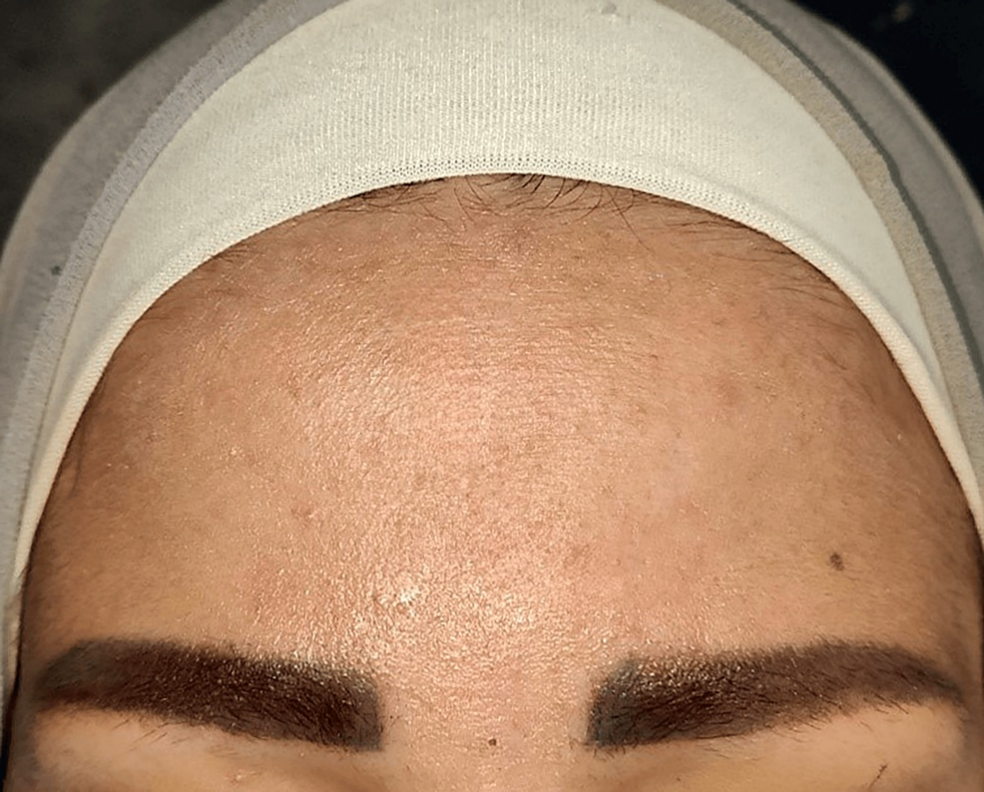
At the six-month follow-up after the procedure

The patient was instructed to apply ice packs to the face for 10 minutes, limit facial movements for 24 hours, avoid facial creams or cosmetics for 48 hours, and avoid pulling or massaging the face for two weeks. It was recommended that pain relievers be taken only as needed if the patient experienced pain. The patient was followed up after six months, and there was visible improvement in the scar (Figure 5).

Case 2

**Figure 6:**
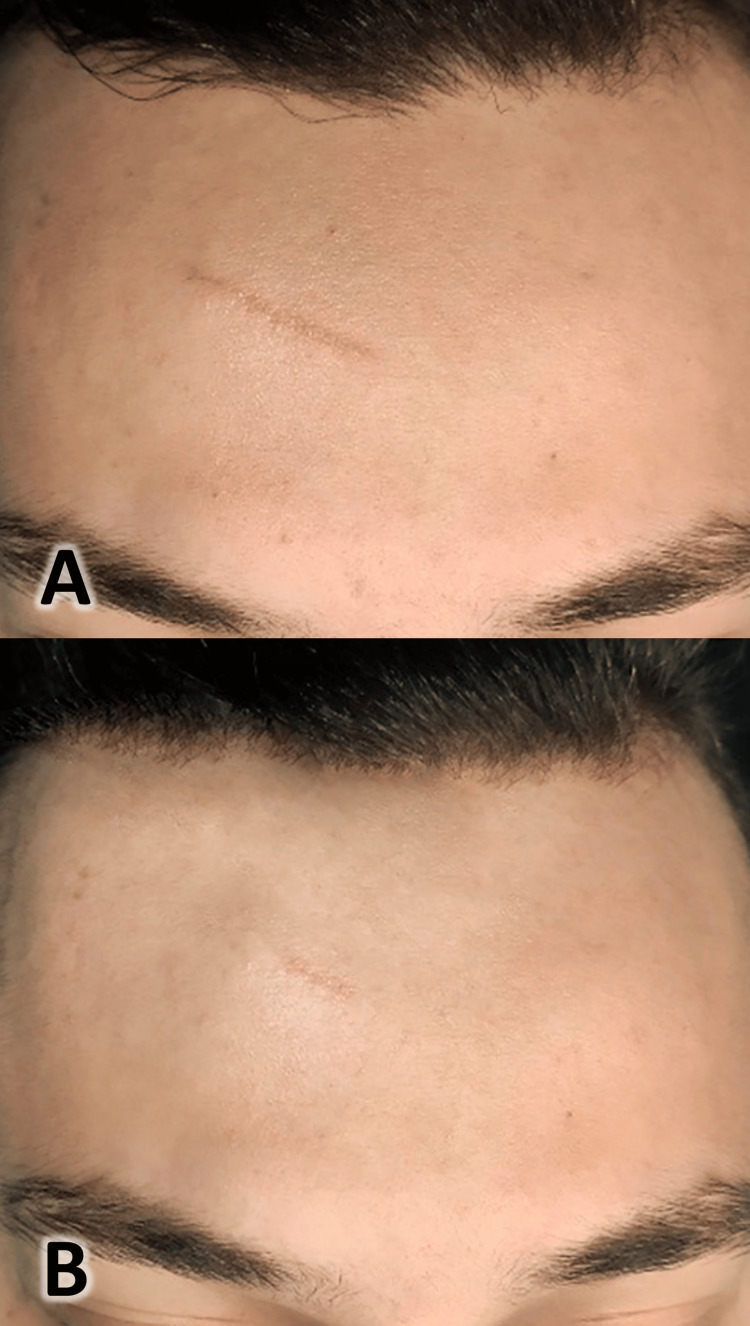
Scar as seen before the procedure (A) and after six months of follow-up (B)

A 27-year-old male patient complained of a facial atrophic scar of nine years, resulting from a laceration injury to the forehead area; two years ago, the patient was offered surgical treatment of the scar (surgical removal of the scar), but he rejected this treatment option because it was invasive. The patient used many cosmetic restorative creams for four months, but he did not notice a change in the appearance of the scar. We offered the patient to treat the scar using polydioxanone threads, and he agreed to it. The treatment was performed using polydioxanone threads, following the same method as described in case 1, after explaining the treatment plan and obtaining the patient's consent. First, the skin was disinfected with a 4% povidone solution, and then benzocaine topical anesthetic and local anesthesia were administered. Isolating the site, a needle was inserted along the scar length, a cannula was inserted and the threads were left in place in the subcutaneous layer; the excess part of the threads was trimmed. The clinical photographs were taken before the procedure and at the six-month follow-up (Figure 6). An improvement in the color of the scar was noticed, as it became more harmonious with the skin tone. Additionally, the scar became more flexible, and its overall size decreased. However, the incomplete disappearance of the scar can be attributed to the fact that this scar was present for nine years before treatment, and it had a relatively dark color from the beginning.

Case 3

**Figure 7:**
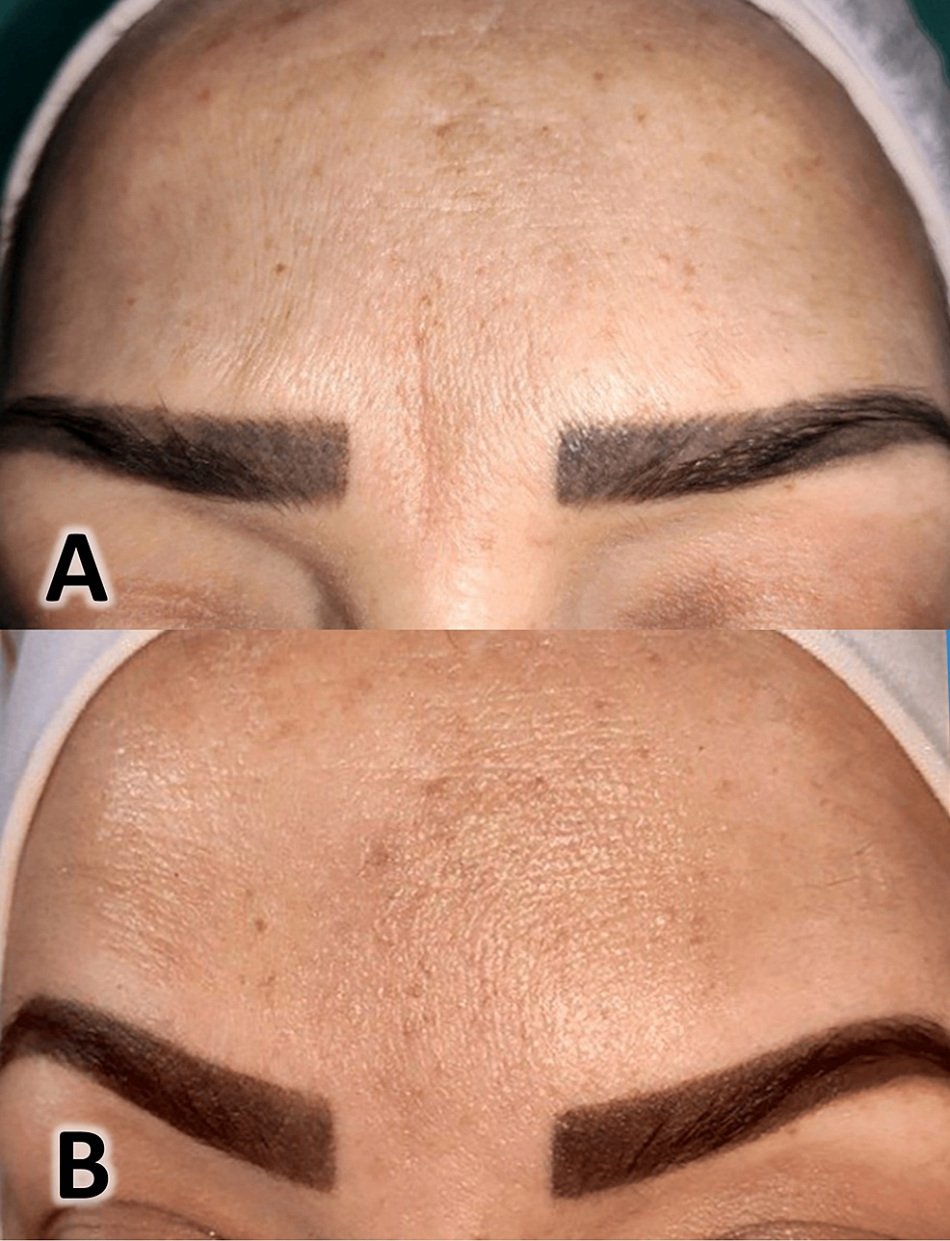
Pictures taken before (A) and after six months of the procedure (B)

A 33-year-old female patient presented with a facial atrophic scar of six years, resulting from a laceration injury between the eyebrows, with no prior treatment for the scar. Previously, the patient was offered treatment using fillers, but she refused it because of the possible complications. She refused to inject autologous fat into the scar area because of the pain and discomfort associated with the liposuction process from the donor site. The treatment was performed using polydioxanone threads following the same method described in case 1 after providing an explanation of the treatment plan and obtaining the patient's consent (skin disinfected with a 4% povidone solution, benzocaine topical anesthetic and local anesthesia administration, surgical site isolation, followed by insertion of a needle along the scar length and cannula insertion, and then threads left in place in the subcutaneous layer and the excess part of the threads trimmed). Clinical photographs were taken before the procedure and at the six-month follow-up, as shown in Figure 7.

## Discussion

Healthy skin is an important barrier against external factors, and the wound-healing process is crucial in restoring tissue stability [1]. Scarring is a part of the healing process, but delays in this process can lead to the formation of undesirable scars, which can impact the psychological well-being of a person, especially when they occur in prominent areas such as the face [8,9]. Treatment techniques for skin scars vary depending on their nature. Treatments that help remove excess tissue, such as topical injections and surgery, are used for hypertrophic scars characterized by elevated surfaces. On the other hand, for atrophic scars characterized by low surfaces, techniques such as dermabrasion and tissue fillers using materials like hyaluronic acid and autologous fat are employed [10,11]. In recent years, highly elastic absorbable threads such as polydioxanone threads have been used to compensate for volume deficits in atrophic scars, with the advantage of their ability to resist forces applied to the skin and stimulate inflammatory and immune responses [12].

PDO threads are available in various commercial forms. They can be smooth, with a surface free from details, or barbed containing microscopic projections that help secure the threads in the targeted tissue site by binding them to the surrounding tissue components [13]. Both types of threads have been used in facial cosmetic treatments, but the smooth form distinguishes itself by its greater ease of application [13]. Additionally, it can fill the targeted tissue while maintaining the integrity of the skin tissues, especially in the facial area. It reduces tissue damage occurring when applying the thread within the targeted tissues. This study used smooth polydioxanone threads, consisting of 14 threads measuring 7/0, threaded onto a needle to penetrate the scar. PDO threads treat atrophic skin scars by applying them within the targeted area using a hollow needle containing the threads in its head. This needle is inserted along the scar until its end. The subcutaneous layer is targeted to stabilize these threads. After ensuring the stability of the threads within the targeted site, the thread-carrying needle is withdrawn, leaving the threads within the scar tissue. These threads substitute for the filling material, allowing an increase in tissue volume to compensate for the deficit caused by the atrophic scar [12,14].

According to this case series report, it was inferred that effective treatment with PDO threads requires careful scar selection. We suggest choosing scars that are atrophic and linear, resulting from a sharp cut, and that have existed for at least six months to ensure they have stabilized. The use of PDO threads is contraindicated in cases where the patient has allergies to the polydioxanone material, active dermatological conditions in the scar area, or uncontrolled chronic diseases such as diabetes or is under treatment with immunosuppressive medications. Using the currently described method has some limitations that must be considered. PDO threads are more effective with atrophic and linear scars, whereas they may be less effective with keloid or hypertrophic scars. Additionally, areas that experience continuous muscle movement may not be suitable for thread use. Furthermore, superficial scars may not require thread usage, whereas deep scars may require additional techniques to achieve the desired results.

A study evaluated the efficacy of poly-L-lactic acid (PLLA) threads in treating atrophic facial scars, comparing them with hyaluronic acid. The threads were used in 40 patients and they were followed up for six months. The study found a clear clinical improvement rate of 82.4% in patients treated with PLLA threads [15]. Researchers also evaluated the effectiveness of PDO threads in treating atrophic scars in the neck area, in a study of 23 patients. PDO threads were used to fill the tissue defect within the scar, and after six months of treatment, improvement was observed in the treated scars, with a positive impact on aesthetics and patient satisfaction. The researchers concluded that using PDO threads improves cosmetic outcomes, promotes scar healing, increases patient satisfaction, and reduces the need for corrective surgery for scar defects [16].

Another study evaluated the effectiveness of PDO monofilament threads compared to carbon dioxide (CO_2_) laser in treating atrophic facial scars. The study included 10 patients, with laser treatment applied to the right side of the face and PDO threads to the left side. Patients were followed up for three months and the treatment response was evaluated using three-dimensional scanning. It was found that both techniques could improve facial aesthetics and skin texture in the treated areas. However, the researchers preferred the CO_2_ laser for its ease of application while noting that PDO threads were less costly for patients [6].

## Conclusions

Polydioxanone threads are considered an easy, available, and biocompatible material. PDO helps stimulate the skin to produce collagen, enhancing skin regeneration and improving scars' appearance over time. PDO threads are biodegradable and absorbable by the body, which reduces the risk of irritation or rejection by the body. They provide natural-looking results by enhancing the skin's natural biological processes without the need for foreign materials or fillers. The presented cases showed a clear improvement in atrophic facial scar management with good patient satisfaction. The treatment of atrophic facial scars using polydioxanone threads is considered a safe and less invasive method which means a shorter recovery period and less pain and side effects compared to other methods (such as surgical treatment or autologous fat injections).
